# Cancer Curers

**Published:** 1869-05-01

**Authors:** 


### EDITORIAL.

### *Cancer Curers.*

An esteemed professional friend in Indiana transmits to us the following note, from an inquiring patient, which contains its own moral:

**SIR:—My wife is afflicted with a cancer on her left side, pretty well up under the arm. She has been under the treatment of a "cancer quack" at**

Chicago for three months, but he has finally given up her case, after receiving a large sum of money from me to warrant a cure. He applied a salve, which eat a great hole in her side; he now refuses to apply the salve to the part affected, on the ground, I believe, that it is too near an artery. I have been advised to write to you, and inquire whether you can do any thing for her.

Please let me know early, as time is precious. Yours Truly, etc.

